# A machine learning-based model for predicting recurrence in intermediate- and high-risk differentiated thyroid cancer: insights from a retrospective single-center study of 2388 patients

**DOI:** 10.3389/fendo.2025.1552479

**Published:** 2025-06-17

**Authors:** Yi Li, Zimei Tang, Anwen Ren, Gang Tian, Jianing Zhang, Yiran Wang, Jie Liu, Jie Ming

**Affiliations:** ^1^ Department of Breast and Thyroid Surgery, Union Hospital, Tongji Medical College, Huazhong University of Science and Technology, Wuhan, China; ^2^ Department of Radiology, Union Hospital, Tongji Medical College, Huazhong University of Science and Technology, Wuhan, China

**Keywords:** differentiated thyroid cancer (DTC), cancer recurrence, predictive models, machine learning, risk factors, random forest

## Abstract

**Purpose:**

Current guidelines provide a recognized yet broad framework for stratifying recurrence risk in differentiated thyroid cancer (DTC) patients. More precise tools are needed for intermediate- and high-risk groups. This study aims to identify recurrence-associated risk factors and develop a machine learning-based predictive model.

**Methods:**

In this retrospective analysis, 2,388 DTC patients were randomly assigned to a training group (1,910 cases) and a validation group (478 cases). Predictive factors were identified using univariate and multivariate analyses. Six machine learning models were trained and validated, with performance evaluated through accuracy, area under the curve, and clinical utility via decision curve analysis.

**Results:**

Independent risk factors for recurrence included intraglandular dissemination, total tumor size, bilateral cervical lymph node involvement, and Hashimoto’s thyroiditis, while normal/elevated TSH and multifocal nodules were protective. The random forest model demonstrated the best performance (training accuracy: 0.801; validation accuracy: 0.808). A random forest-based online calculator was developed to facilitate individualized risk assessment in clinical settings.

**Conclusions:**

The random forest model effectively predicts DTC recurrence, offering a practical tool for individualized risk assessment and aiding clinical decision-making.

## 1 Introduction

Differentiated thyroid cancer (DTC), primarily comprising papillary and follicular subtypes, is the most common endocrine malignancy, accounting for approximately 90% of thyroid cancer cases (1). Despite its generally favorable prognosis, with a five-year survival rate exceeding 95%, a subset of DTC patients experiences a higher probability of recurrence, which significantly impacts long-term health outcomes (2).

The 2015 American Thyroid Association (ATA) guidelines offer a widely recognized framework for stratifying the risk of recurrence in DTC patients based on factors such as tumor size, histopathological characteristics, and the presence of metastases. Patients are classified into low-, intermediate-, and high-risk groups, with recurrence rates ranging from 3–13% in low-risk, 21–36% in intermediate-risk, and approximately 68% in high-risk patients (3). While these guidelines are broadly effective, their ability to accurately predict individual recurrence risks remains limited, especially for intermediate- and high-risk groups (4). This limitation is primarily due to the limited number of factors included and the equal weighting assigned to each, thereby hindering personalized clinical management.

In recent years, machine learning (ML) has emerged as a powerful tool for analyzing large, complex healthcare datasets (5). Unlike traditional statistical methods, ML algorithms excel at processing high-dimensional data and identifying intricate non-linear relationships, thereby enhancing predictive accuracy in oncology (6). Numerous studies have demonstrated the efficacy of ML models in survival prediction and recurrence monitoring in various cancer types, promoting a more personalized and data-driven approach to patient management (7). However, their application in refining risk stratification for DTC, particularly within intermediate- and high-risk cohorts, remains underexplored.

Addressing this gap, our study aims to develop and validate an ML-based recurrence prediction model tailored for intermediate- and high-risk DTC patients. Utilizing a robust retrospective cohort of 2,388 DTC patients from our center, we trained and validated multiple ML algorithms on demographic, clinical, and pathological features to predict recurrence. Model performance was assessed through metrics such as the area under the receiver operating characteristic curve (AUC), sensitivity, and specificity. By enhancing the precision of recurrence risk assessment, the proposed model facilitates more individualized and effective clinical management for intermediate- and high-risk DTC patients.

## 2 Materials and methods

### 2.1 Population and data collection

We retrospectively retrieved clinical records of DTC patients treated between 2009 and 2021 at the Department of Breast and Thyroid Surgery, Union Hospital, Tongji Medical College, Huazhong University of Science and Technology (WHUH). The data were used to establish training and validation cohorts. Patients were included if they met the following criteria: (1) pathologically confirmed DTC; (2) underwent total thyroidectomy or lobectomy; and (3) classified as having intermediate or high recurrence risk according to the 2015 ATA guidelines (3). Exclusion criteria were: (1) history of other malignancies and (2) incomplete clinical records. Only patients who underwent reoperation and had DTC recurrence confirmed by pathology was defined as recurrence. Our study adopted pathologically confirmed thyroid carcinoma as the objective criterion for reoperation grouping, primarily due to inherent limitations in postoperative surveillance completeness within retrospective data - specifically, the absence of consecutive ultrasound or thyroglobulin monitoring records in some patients precluded precise definition of a ‘disease-free interval’.

Our database retrospectively collected demographic information, ultrasound (US) findings, biochemical test results, and pathological characteristics of the enrolled patients. Preoperative US identified the size, location, number, echogenicity, calcifications, cervical lymph nodes (LNs), and extrathyroidal extension (ETE) of nodules. Clinically evident metastatic lymph nodes (cN1) were defined by features such as calcifications, loss of fatty hilum, disrupted medullary architecture, and cystic changes on US (3, 8, 9). Bilateral and multifocal lesions, as confirmed by US and intraoperative findings, referred to disease affecting both thyroid lobes and the presence of two or more foci in one or both lobes, respectively. Palpable nodules and LNs were those detectable on preoperative physical examination.

Hashimoto’s thyroiditis (HT) was confirmed by intraoperative frozen sections characterized by diffuse infiltration of lymphocytes and plasma cells, the formation of lymphoid follicles with germinal centers within the gland, fibrosis, and atrophy of thyroid parenchyma (10, 11). Postoperative pathology determined the number, size, distribution, subtype, invasiveness of cancer foci, as well as the number and location of metastatic LNs. The total tumor size was calculated as the sum of the maximum diameters of all excised cancer foci.

### 2.2 Statistical analysis

Missing values were handled using the Multiple Imputation by Chained Equations (MICE) method, with appropriate imputation algorithms selected based on variable type (12, 13). Predictive mean matching (PMM) was applied for continuous variables, logistic regression (LogReg) for binary variables (14), and polynomial regression (PolyReg) for categorical variables with more than two levels (15). The imputed datasets were used for subsequent analyses.

For continuous variables, the Shapiro-Wilk test was employed to assess their normality. Variables following a normal distribution were analyzed using independent-sample t-tests to evaluate their associations with the outcome variable, while non-normally distributed variables were assessed with the Mann-Whitney U test. Categorical variables were analyzed using the chi-squared test or Fisher’s exact test, depending on cell frequencies in contingency tables.

Multivariate analysis was conducted using stepwise logistic regression based on the Akaike Information Criterion (AIC) to identify the optimal model for evaluating factors associated with the outcome variable. Model fit was assessed using the Hosmer-Lemeshow test, while discriminatory performance was evaluated with Receiver Operating Characteristic (ROC) curves and AUC values.

The final multivariate model included the following predictors: total tumor size, HT, lateral cervical lymph node metastasis (LLNM), intraglandular dissemination, the number of nodules >1 cm identified on preoperative US, palpable nodules, nodule texture, nodule calcification on US, multifocality on US, size of lymph node area with suspicion of metastasis identified preoperatively, preoperative TSH levels, and central LN metastasis (CLNM).

Odds ratios (OR) and their corresponding 95% confidence intervals (CI) were calculated for both categorical and continuous variables using logistic regression. Data analyses were performed using R software (version 4.4.1).

### 2.3 Development and comparison of ML-based models

The use of the Random Over-Sampling Examples (ROSE) method was necessitated by the severe class imbalance in the dataset, where recurrence cases (minority class) were underrepresented. Traditional models trained on such data often prioritize majority-class accuracy, leading to poor sensitivity for recurrence prediction—a critical shortcoming in clinical settings. ROSE was chosen over alternatives like SMOTE due to its ability to generate synthetic minority samples using bootstrapping and kernel density estimation, introducing controlled noise to simulate realistic feature variations. This approach avoids deterministic interpolation, which risks overfitting by creating artificial linear patterns, while expanding the diversity of the minority class.We used cross-validation to avoid the problem of overfitting.

For model development and validation, the dataset was randomly split into a training cohort (80%) and a validation cohort (20%). Six popular ML models—K-nearest neighbors (KNN), decision trees (DT), support vector machines (SVM), extreme gradient boosting (XGBoost), logistic regression (LR), and random forest (RF)—were trained using significant predictors identified in multivariate analyses.

The models’ performance was evaluated using multidimensional metrics, including accuracy, AUC, sensitivity, specificity, false positive rate (FPR), and false negative rate (FNR). Higher values for accuracy, AUC, sensitivity, and specificity indicate better performance, while lower FPR and FNR are desirable. Decision curve analysis (DCA) was conducted to assess the clinical utility of the models by estimating net benefits at various threshold probabilities. DCA calculates the net benefit of treating patients within a specific threshold probability, balancing true-positive benefits against false-positive harms (16).

To enhance interpretability, feature importance analysis was performed to evaluate the contribution of variables to the models. Feature importance quantifies the impact of individual predictors by measuring the increase in model prediction error after permuting each feature. This approach helps identify variables with the greatest influence on predictive outcomes.

### 2.4 Model validation and web development

Following the selection of the best-performing model, internal validation was conducted using the reserved validation cohort. The same evaluation metrics employed for model comparison were applied to assess performance, including accuracy, AUC, sensitivity, specificity, FPR, and FNR. A confusion matrix was generated to illustrate discrepancies between actual and predicted outcomes. Calibration curves were constructed to evaluate the agreement between predicted probabilities and observed outcomes, providing insight into the model’s reliability.

To facilitate clinical application, a web-based calculator was developed using the R package Shiny. This tool allows clinicians to input patient-specific data and obtain individualized recurrence risk predictions based on the developed ML model.

## 3 Results

### 3.1 Clinical characteristics

**Figure 1:**
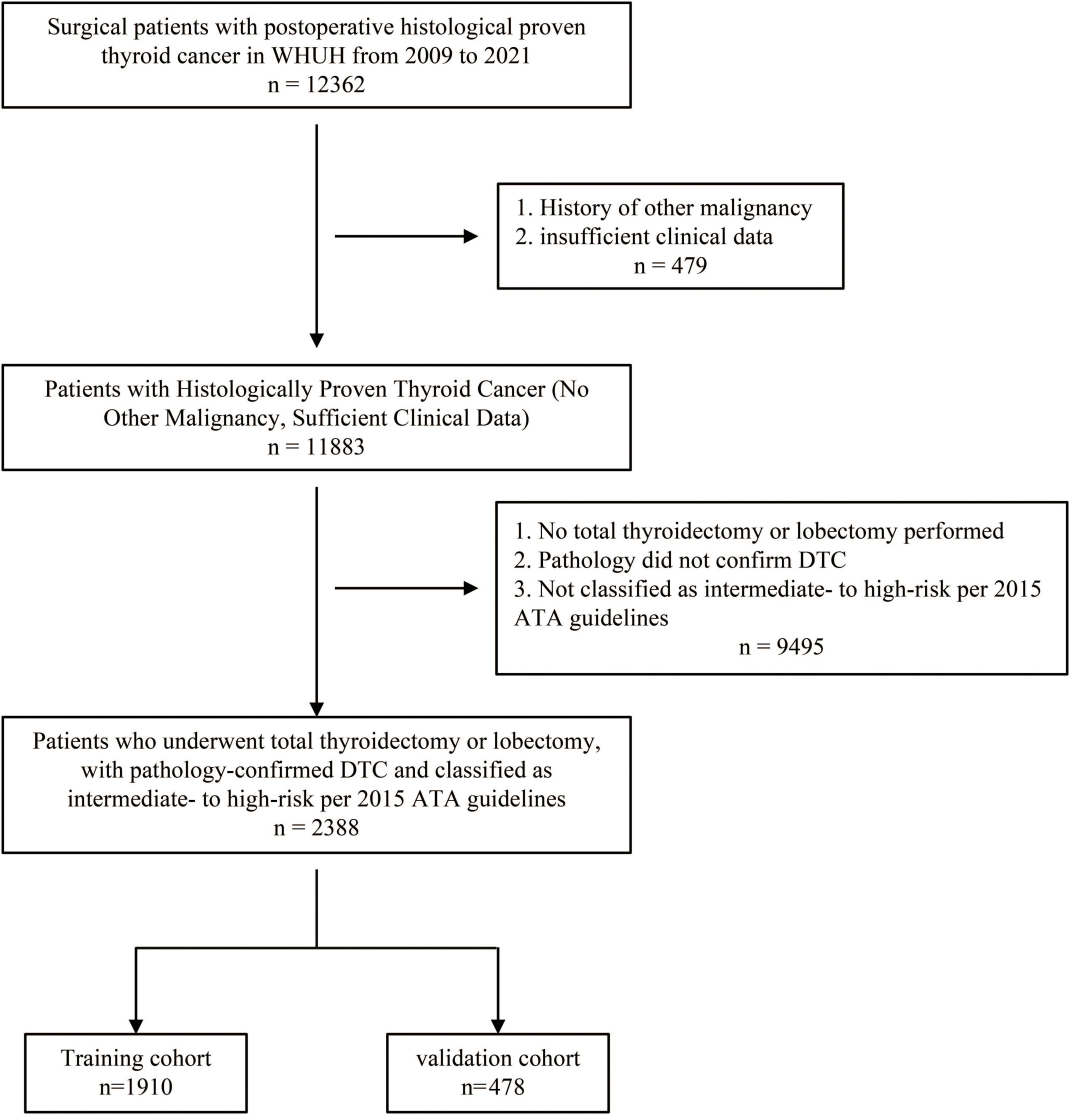
Flowchart of patient selection. A total of 12,362 individuals who underwent thyroid surgery were identified from the WHUH database. After applying inclusion and exclusion criteria, 2,388 DTC patients were included in the study. DTC, differentiated thyroid cancer; WHUH, Union Hospital, Tongji Medical College, Huazhong University of Science and Technology.

A total of 2,388 patients were included in this study, selected from the WHUH database comprising 12,362 individuals who underwent thyroid surgery (**Figure 1**). Among the cohort, 139 patients (5.82%) experienced recurrence during follow-up, while 2,249 (94.18%) did not. **Table 1** summarizes of the demographic and clinicopathological characteristics of the cohort.

**Table 1:** Demographic and clinicopathologic features of the patients grouped by recurrence.

Variable	Levels	Total (n=2388)	Non-recurrence (n=2249)	Recurrence (n=139)	P-value
		2388	2249	139	
Age (%)	<=55y	2107(88.23%)	1980(88.04%)	127(91.37%)	0.296
	>55y	281(11.77%)	269(11.96%)	12(8.63%)	
Gender (%)	Female	1678(70.27%)	1588(70.61%)	90(64.75%)	0.170
	Male	710(29.73%)	661(29.39%)	49(35.25%)	
Marital status (%)	No	368(15.41%)	341(15.16%)	27(19.42%)	0.320
	Yes	2008(84.09%)	1896(84.3%)	112(80.58%)	
	Others	12(0.5%)	12(0.53%)	0(0%)	
BMI (%)	Low	121(5.07%)	116(5.16%)	5(3.6%)	0.507
	Normal	1578(66.08%)	1489(66.21%)	89(64.03%)	
	High	689(28.85%)	644(28.63%)	45(32.37%)	
Family history (%)	No	2346(98.24%)	2212(98.35%)	134(96.4%)	0.094
	Yes	42(1.76%)	37(1.65%)	5(3.6%)	
Hypertensive (%)	No	2258(94.56%)	2127(94.58%)	131(94.24%)	1.000
	Yes	130(5.44%)	122(5.42%)	8(5.76%)	
Diabetes (%)	No	2355(98.62%)	2216(98.53%)	139(100%)	0.258
	Yes	33(1.38%)	33(1.47%)	0(0%)	
Hyperthyroidism (%)	No	2357(98.7%)	2219(98.67%)	138(99.28%)	1.000
	Yes	31(1.3%)	30(1.33%)	1(0.72%)	
HT (%)	No	2367(99.12%)	2234(99.33%)	133(95.68%)	<0.001
	Yes	21(0.88%)	15(0.67%)	6(4.32%)	
FT3 (%)	Low	26(1.09%)	24(1.07%)	2(1.44%)	0.672
	Normal	2292(95.98%)	2160(96.04%)	132(94.96%)	
	High	70(2.93%)	65(2.89%)	5(3.6%)	
FT4 (%)	Low	86(3.6%)	79(3.51%)	7(5.04%)	0.213
	Normal	2228(93.3%)	2103(93.51%)	125(89.93%)	
	High	74(3.1%)	67(2.98%)	7(5.04%)	
Preoperative TSH levels (%)	Low	104(4.36%)	79(3.51%)	25(17.99%)	<0.001
	Normal	2193(91.83%)	2081(92.53%)	112(80.58%)	
	High	91(3.81%)	89(3.96%)	2(1.44%)	
Palpable nodule (%)	No	740(30.99%)	686(30.5%)	54(38.85%)	<0.05
	Yes	1648(69.01%)	1563(69.5%)	85(61.15%)	
Palpable LN (%)	No	1942(81.32%)	1828(81.28%)	114(82.01%)	0.918
	Yes	446(18.68%)	421(18.72%)	25(17.99%)	
Nodule texture (%)	Soft	219(9.17%)	196(8.71%)	23(16.55%)	<0.05
	Mediun	378(15.83%)	356(15.83%)	22(15.83%)	
	Hard	1791(75%)	1697(75.46%)	94(67.63%)	
FNA (%)	No	2064(86.43%)	1934(85.99%)	130(93.53%)	<0.05
	Yes	324(13.57%)	315(14.01%)	9(6.47%)	
US-detected bilateral lesions (%)	No	1523(63.78%)	1426(63.41%)	97(69.78%)	0.153
	Yes	865(36.22%)	823(36.59%)	42(30.22%)	
US-detected multifocality (%)	No	1461(61.18%)	1361(60.52%)	100(71.94%)	<0.05
	Yes	927(38.82%)	888(39.48%)	39(28.06%)	
Regions of lymph node with suspicion of metastasis on US (%)	No	1351(56.57%)	1273(56.6%)	78(56.12%)	<0.05
	Central LN	228(9.55%)	216(9.6%)	12(8.63%)	
	Cervical LN	637(26.68%)	607(26.99%)	30(21.58%)	
	Central LN +cervical LN	106(4.44%)	93(4.14%)	13(9.35%)	
	Central LN +bilateral cervical LN	66(2.76%)	60(2.67%)	6(4.32%)	
US-detected capsular invasion (%)	No	2338(97.91%)	2201(97.87%)	137(98.56%)	1.000
	Yes	50(2.09%)	48(2.13%)	2(1.44%)	
cN1 (%)	No	1392(58.29%)	1311(58.29%)	81(58.27%)	1.000
	Yes	996(41.71%)	938(41.71%)	58(41.73%)	
ETE (%)	No	2070(86.68%)	1959(87.11%)	111(79.86%)	<0.05
	Yes	318(13.32%)	290(12.89%)	28(20.14%)	
High-risk subtype (%)	No	2258(94.56%)	2122(94.35%)	136(97.84%)	0.083
	Yes	130(5.44%)	127(5.65%)	3(2.16%)	
Bilateral lesions (%)	No	1418(59.38%)	1343(59.72%)	75(53.96%)	0.210
	Yes	970(40.62%)	906(40.28%)	64(46.04%)	
Involvement of the thyroid isthmus (%)	No	2301(96.36%)	2169(96.44%)	132(94.96%)	0.173
	Yes	83(3.48%)	77(3.42%)	6(4.32%)	
	Only isthmus	4(0.17%)	3(0.13%)	1(0.72%)	
Intraglandular dissemination (%)	No	1484(62.14%)	1436(63.85%)	48(34.53%)	<0.001
	Yes	904(37.86%)	813(36.15%)	91(65.47%)	
maximum tumor size (%)	≧1cm	944(39.53%)	902(40.11%)	42(30.22%)	<0.05
	≦4cm	1376(57.62%)	1287(57.23%)	89(64.03%)	
	>4cm	68(2.85%)	60(2.67%)	8(5.76%)	
CLNM (%)	No	494(20.69%)	455(20.23%)	39(28.06%)	<0.05
	Yes	1894(79.31%)	1794(79.77%)	100(71.94%)	
LLNM (%)	No	1392(58.29%)	1328(59.05%)	64(46.04%)	<0.05
	Yes	996(41.71%)	921(40.95%)	75(53.96%)	
US-detected nodule count (mean (SD))		2.19(1.12)	2.19(1.12)	2.12(1.15)	0.286
US-detected >1cm nodule count (mean (SD))		1.01(0.76)	1(0.76)	1.16(0.8)	<0.05
US-detected calcified nodule		0.93(0.91)	0.94(0.9)	0.79(1.02)	<0.05
US-detected LN with suspicion of metastasis (mean (SD))		0.72(1.05)	0.71(1.03)	0.82(1.3)	0.645
Number of mixed-echogenic nodules (mean (SD))		0.03(0.19)	0.03(0.2)	0.01(0.12)	0.349
Number of solid hypoechoic nodules		0.66(1.09)	0.67(1.09)	0.54(1.14)	<0.05
Total tumor size (mm, mean (SD))		18.84(13.81)	18.63(13.6)	22.21(16.59)	<0.05
CLNR (mean (SD))		0.41(0.3)	0.41(0.3)	0.44(0.33)	0.282
LLNR (mean (SD))		0.26(0.23)	0.26(0.23)	0.27(0.24)	0.849
LNR (mean (SD))		0.31(0.27)	0.31(0.27)	0.32(0.23)	0.877

BMI, Body Mass Index; FT3, Free Triiodothyronine; FT4, Free Thyroxine; CLNM, Central Lymph Node Metastases; LLNM, Lateral Cervical Lymph Node Metastases; CLNR, Central Lymph Node Ratio; LLNR, Lateral Cervical Lymph Node Ratio; LNR, Lymph Node Ratio; Normal-BMI (18.0<= BMI <25.0), Low-BMI (BMI <18.0), and High-BMI (BMI >=25.0); Normal TSH level: 0.35–4.94 μIU/ml; Normal FT3 level: 2.63–5.70 pmol/L; Normal FT4 level: 9.00–19.18 pmol/L; FNA (%) indicates the proportion of patients undergoing preoperative fine-needle aspiration.

### 3.2 Feature selection

**Figure 2:**
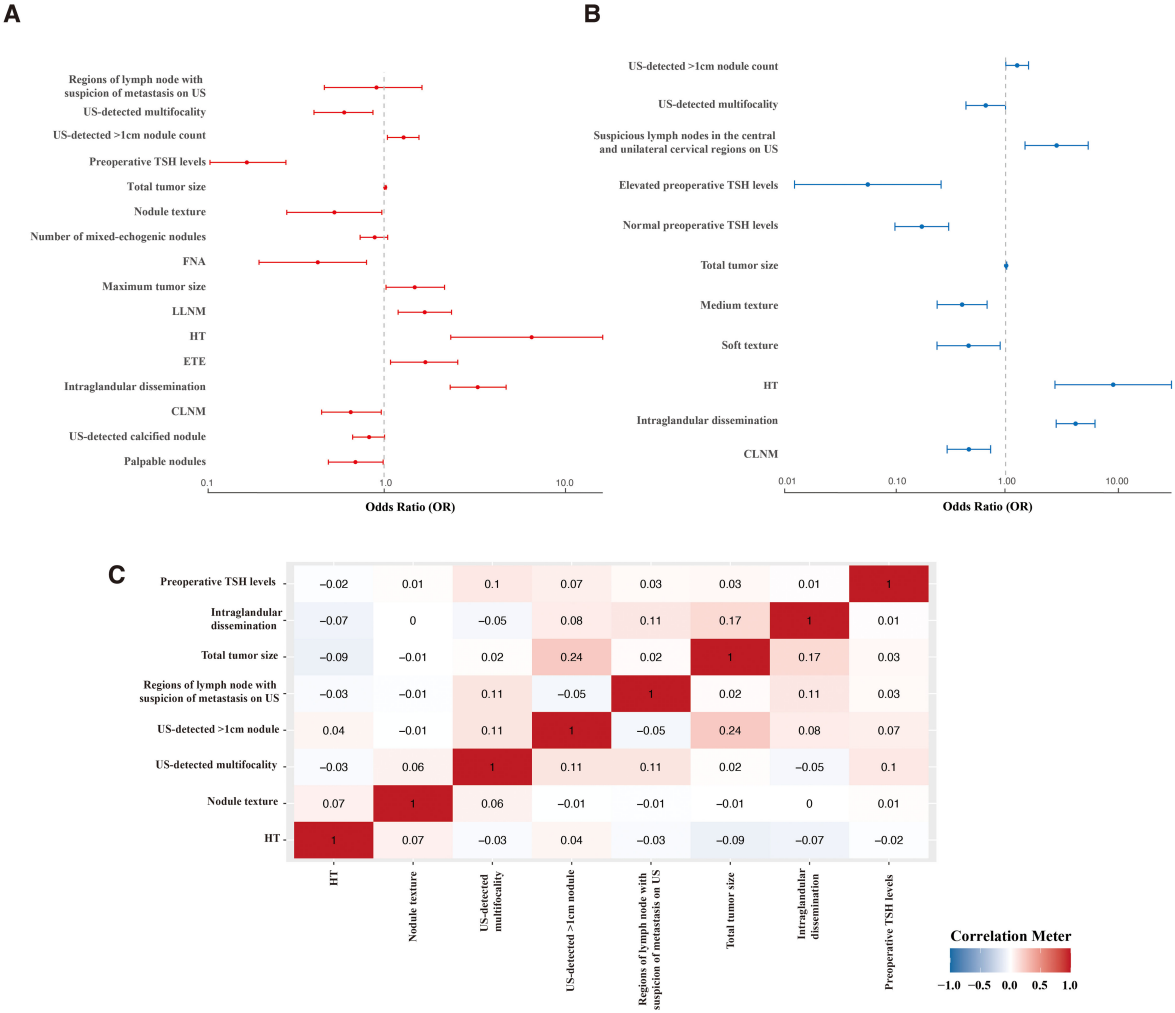
Feature selection. Forest plot of univariate **(A)** and multivariate **(B)** analyses identifying factors predicting recurrence. **(C)** Correlation analysis of selected factors. FNA, Fine-Needle Aspiration; HT, Hashimoto’s thyroiditis; ETE, extrathyroidal extension; LLNM, lateral cervical lymph node metastasis; CLNM, central lymph node metastasis.

Univariate analysis identified significant associations between recurrence and several variables, including maximum tumor size, total tumor size, HT, ETE, LLNM, intraglandular dissemination, US-detected >1cm nodule count, palpable nodules, nodule texture, US-detected calcified nodule, mixed echogenicity nodules on US, US- detected mutifocality, size of lymph node area with suspicion of metastasis on US, fine-needle aspiration (FNA), preoperative TSH levels, and CLNM (P < 0.05) (**Figure 2A**).

In multivariate analysis, independent risk factors for recurrence included intraglandular dissemination (OR = 4.347, 95% CI: 2.894–6.529, P < 0.001), total tumor size (OR = 1.012, 95% CI: 1.000–1.025, P < 0.05), suspicious LNs in the central and bilateral cervical regions on US (OR = 2.919, 95% CI: 1.504–5.668, P < 0.001), US-detected >1cm nodule count (OR = 1.275, 95% CI: 1.004–1.620, P < 0.05), and HT (OR = 9.575, 95% CI: 2.819–32.525, P < 0.001).

Protective factors included soft nodule texture (OR = 0.460, 95% CI: 0.237–0.893, P < 0.05) and medium texture (OR = 0.401, 95% CI: 0.238–0.678, P < 0.001), normal (OR = 0.172, 95% CI: 0.098–0.303, P < 0.001) or elevated preoperative TSH levels (OR = 0.055, 95% CI: 0.012–0.258, P < 0.001), CLNM (OR = 0.463, 95% CI: 0.293–0.730, P < 0.001), and US-detected mutifocality (OR = 0.659, 95% CI: 0.435–0.999, P < 0.05) (**Figure 2B**; **Supplementary Table S1**).

The correlation analysis (**Figure 2C**) demonstrated that none of the features had a significant correlation with one another (<0.3). Considering their clinical relevance, all the above factors were included in the ML model.

### 3.3 Machine Learning Model Performance

**Figure 3:**
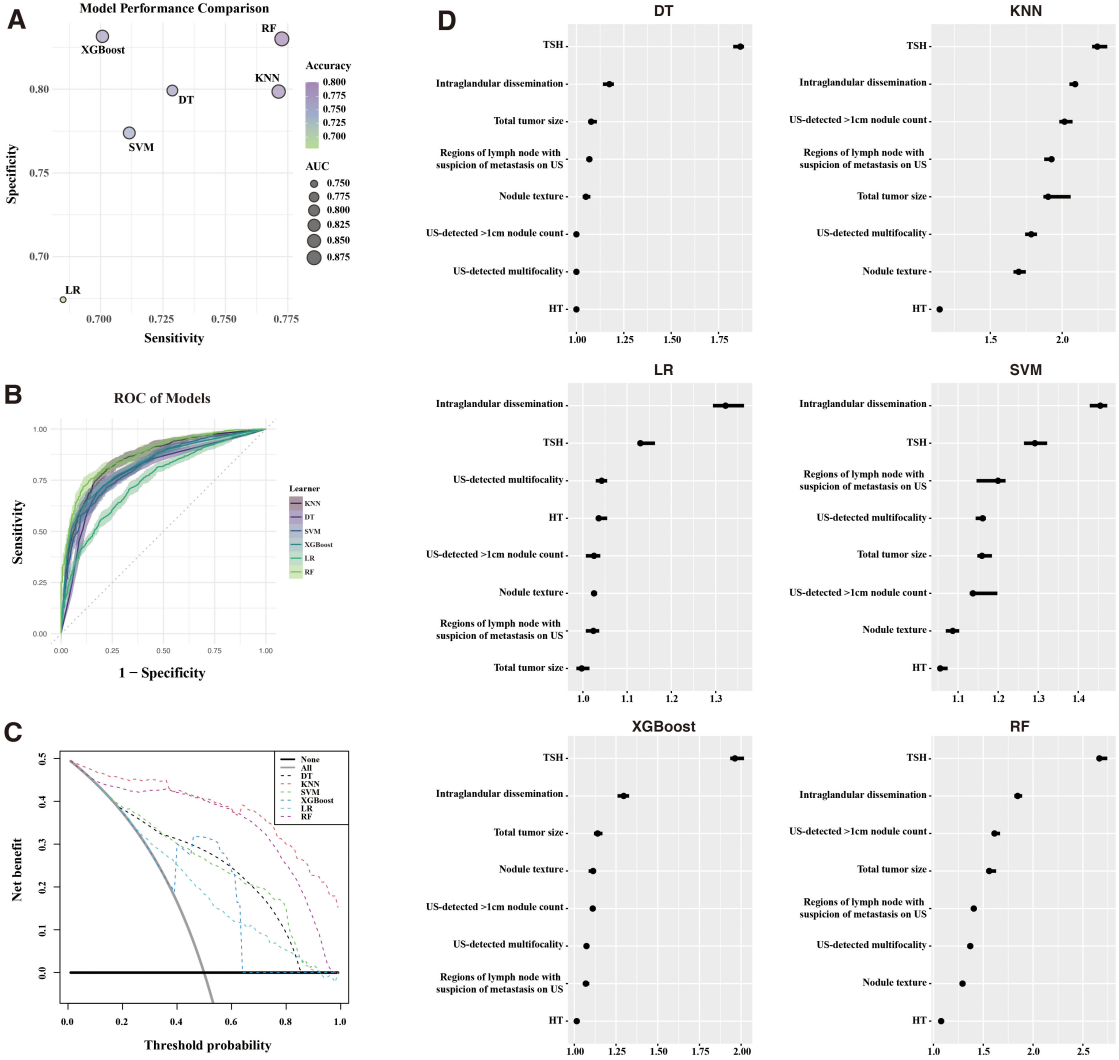
Machine Learning model performance. **(A)** Comparison of sensitivity, specificity, accuracy, and AUC across six ML models. **(B)** ROC curves for each ML model. **(C)** Decision curve analysis (DCA) illustrating clinical benefit of the models. **(D)** Feature importance of models built with recurrence for the six models. ROC, receiver operating characteristic; KNN, K-nearest neighbors; DT, decision trees; SVM, support vector machines; XGBoost, extreme gradient boosting; LR, logistic regression; RF, random forest; AUC, area under the curve.

Using the identified features, six ML models—KNN, DT, SVM, XGBoost, LR, and RF—were developed to predict recurrence. These models were evaluated in the training cohort (**Figures 3A, B**). **Supplementary Table S2** detailed the models. All demonstrated satisfactory performance, with RF achieving the highest accuracy (0.801) and the largest AUC.

DCA (**Figure 3C**) demonstrated that RF and KNN provided the greatest net clinical benefit across threshold probabilities. Feature importance analysis (**Figure 3D**) highlighted the preoperative TSH levels and intraglandular dissemination as the most influential predictors across all models.

### 3.4 RF model validation

**Figure 4:**
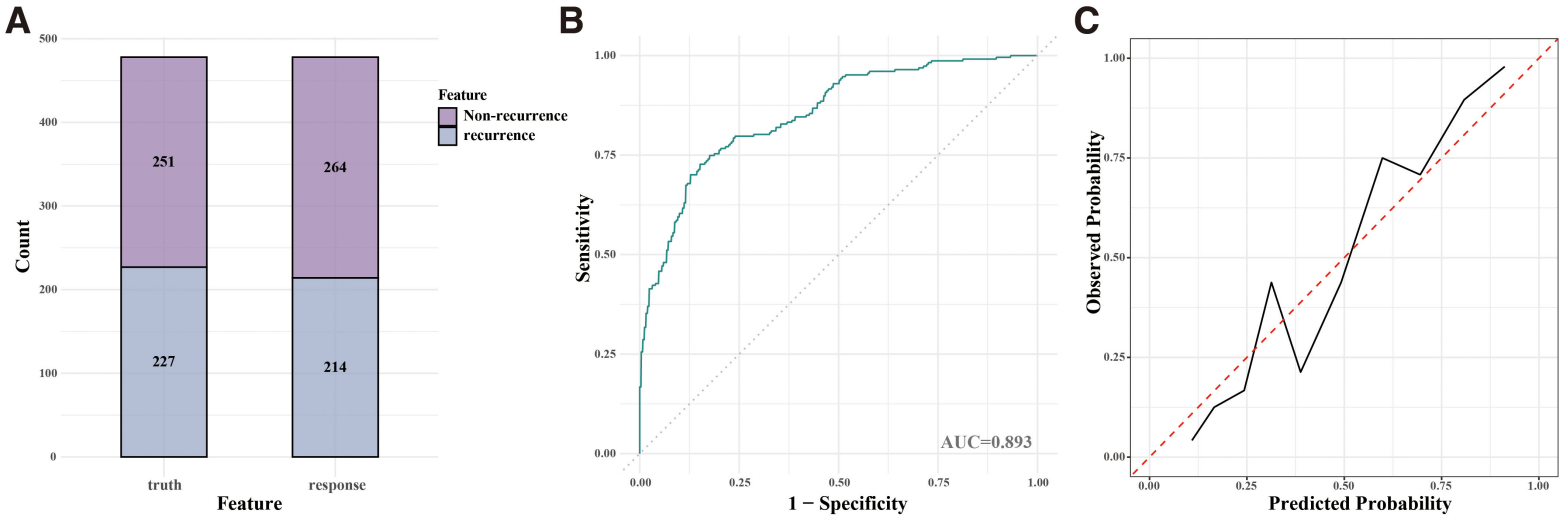
Performance of the Random Forest (RF) model on validation cohorts. **(A)** Confusion matrix for the internal validation cohort. **(B)** ROC curve for the internal validation cohort. **(C)** Calibration curve for the internal validation cohort.

The RF model was validated in the test cohort, where it achieved an accuracy of 0.808, an AUC of 0.893, a sensitivity of 0.776, and a specificity of 0.841. The confusion matrix (**Figure 4A**) demonstrated the model’s performance, and the ROC curve (**Figure 4B**) confirmed its strong discriminative ability. The calibration curve (**Figure 4C**) indicated good agreement between the predicted and observed recurrence probabilities.

### 3.5 Web-based calculator

**Figure 5:**
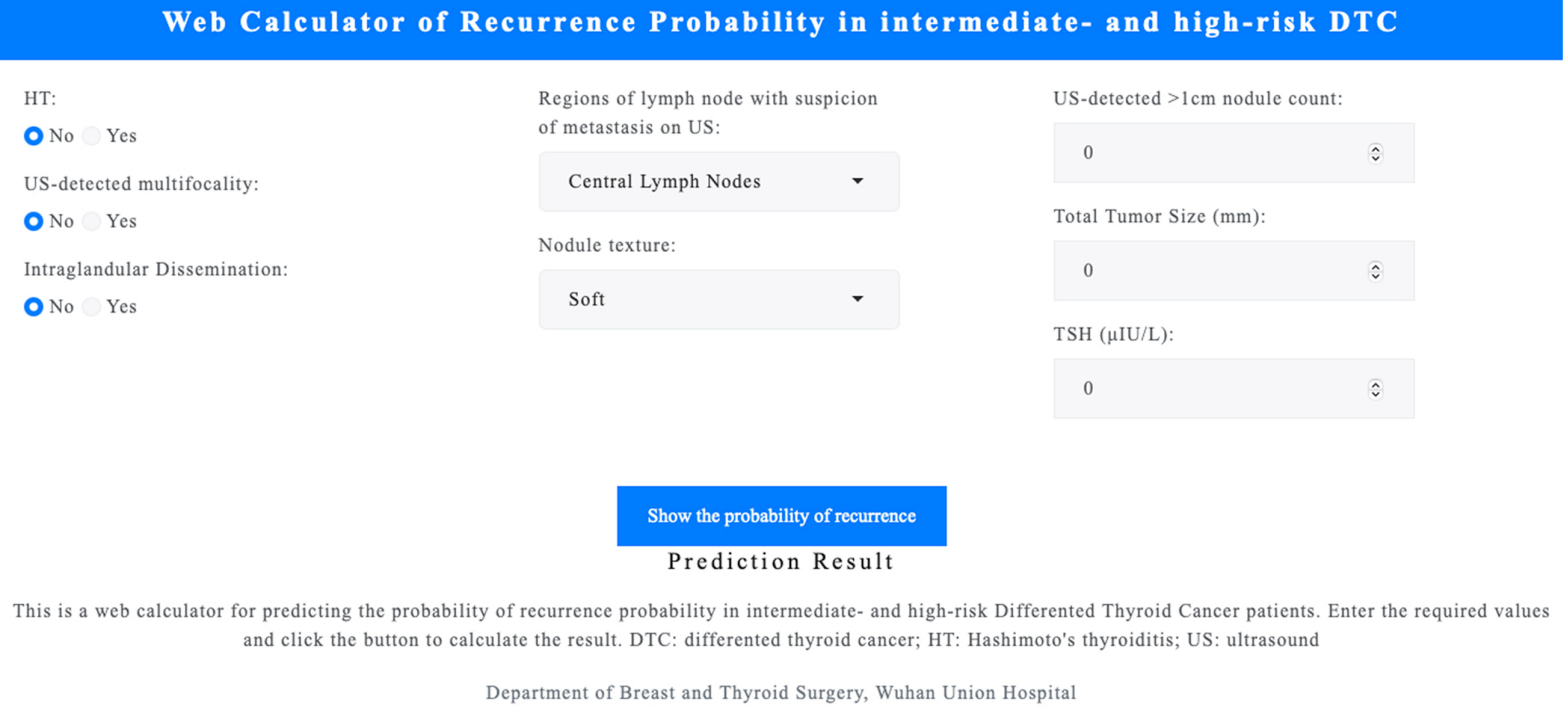
Web-based calculator for recurrence prediction. An interactive tool developed using the R Shiny package allows clinicians to input eight variables to estimate DTC recurrence risk. Accessible at https://leekeeee.shinyapps.io/DTC_Recurrence_Prediction_Model/. DTC, differentiated thyroid cancer; TSH, thyroid-stimulating hormone; LN, lymph node.

An interactive web calculator (https://leekeeee.shinyapps.io/DTC_Reccurence_Prediction_Model/) was developed using the R Shiny package to facilitate clinical application of the RF model. The calculator allows clinicians to input eight variables, including HT, US-detected mutifocality, intraglandular dissemination, regions of lymph node with suspicion of metastasis on US, nodule texture, US-detected >1cm nodule count, total tumor size, and preoperative TSH levels, to estimate recurrence risk (**Figure 5**). This tool provides an accessible means of supporting personalized management of patients with DTC.

## 4 Discussion

This study, based on a comprehensive retrospective analysis of 2,388 DTC patients, identifies and validates risk and protective factors for recurrence. Guided by two criteria—(1) low multicollinearity (pairwise correlation coefficients <0.3, ensuring statistical independence) and (2) clinical relevance rooted in thyroid pathology evidence—our findings reaffirm established predictors (e.g., intraglandular dissemination) while uncovering novel associations (e.g., CLNM as a protective factor). Despite initial counterintuitive trends, these inclusions minimized redundancy and enhanced model robustness, ultimately enriching mechanistic insights into DTC recurrence.

Among the risk factors, intraglandular dissemination emerged as the most significant, reflecting the heightened invasive and metastatic potential associated with tumor cell spread within thyroid tissues. This finding highlights the necessity of meticulous surgical and pathological evaluations to identify and manage these disseminated foci (17–19). Moreover, the association between HT and recurrence risk supports the hypothesis that chronic inflammation fosters a pro-tumorigenic microenvironment (11, 20–26), likely mediated by inflammatory cytokines and immune cell infiltration (24, 27). Such mechanisms may promote tumor proliferation and invasion, warranting further exploration of inflammatory biomarkers in recurrence prediction. Total tumor size, as an indicator of tumor burden, underscores the increased likelihood of residual disease and metastasis, corroborating prior findings (28–30). Conversely, the appearance of softer or moderately textured nodules was observed to confer a protective effect, which may indicate less aggressive tumor behavior or benign pathological characteristics.

Additionally, imaging features played a pivotal role in recurrence prediction. Parameters such as the number of nodules >1 cm (3, 31, 32) and the size of suspicious metastatic LN regions highlight the relevance of comprehensive preoperative evaluation. These findings underscore the complex relationship between US characteristics and recurrence risk assessment (33, 34), suggesting the potential for US data to enhance the precision of imaging-based scoring systems. Notably, patients with US-detected multifocality were significantly more likely to undergo total thyroidectomy (90.23% vs. 84.49% in unifocal cases, P < 0.001)—an aggressive surgical approach that likely reduced residual disease risk. This clinical decision may have artifactually contributed to the observed “protective” association of multifocality in our model, illustrating how treatment patterns can indirectly shape predictive outcomes.

A particularly novel finding of this study is the protective role of normal or elevated preoperative TSH levels against recurrence. While this observation establishes a relationship between TSH levels and recurrence risk, it is inconsistent with most existing studies (21, 35–37), which often associate higher TSH levels with increased tumor aggressiveness or recurrence likelihood. The discrepancy in findings may be attributed to variations in study design, patient population characteristics, or other factors. Specifically, patients with lower baseline TSH might not have received more aggressive TSH suppression therapy (e.g., high-dose thyroid hormone replacement or targeted pharmacological interventions), potentially reflecting a clinical preference for conservative management in perceived low-risk cohorts rather than an intrinsic biological effect of TSH levels.

Another intriguing result is the identification of CLNM as a protective variable. This may be attributed to the extensive surgical clearance often performed in cases with central LN involvement, thereby reducing the residual tumor burden and potentially lowering recurrence risk. Alternatively, it could reflect a bias introduced by the closer postoperative monitoring and tailored treatment strategies these patients may receive. However, given that this finding contradicts the usual understanding of LN metastasis as a risk factor (38–41), it underscores the need for further investigation through larger, multicentric datasets to confirm its validity and clarify its clinical implications. The biological mechanisms underlying these paradoxical associations remain uncertain, highlighting the imperative for mechanistic investigations to differentiate between treatment-driven biases and authentic disease prognosis pathways.

From a methodological standpoint, the RF model demonstrated superior predictive performance compared to other ML algorithms. With AUC values of 0.875 and 0.893 in the training and validation cohorts, respectively, the RF model proved highly effective in recurrence prediction. Notably, clinical utility analysis further highlighted RF’s superiority across various decision thresholds. Among the features contributing most significantly to the model’s performance, preoperative TSH levels and intraglandular dissemination stood out, reflecting their clinical relevance and potential as actionable targets in recurrence prevention strategies.

Despite its strengths, this study has several limitations. As a single-center retrospective analysis, it is susceptible to selection bias, limiting the generalizability of the findings. Due to retrospective data limitations, detailed post-surgical management variables (e.g., RAI dosage, TSH suppression intensity) were not available for analysis, which may confound the interpretation of recurrence predictors. Additionally, while data resampling techniques were employed to address class imbalance, external validation in multicentric cohorts is essential to confirm these results. Moreover, the absence of molecular biomarkers (e.g., BRAF, TERT) and advanced radiomic data in the current analysis limits the model’s precision and applicability. Future research should integrate these dimensions to enhance predictive accuracy and uncover deeper biological insights into recurrence mechanisms.

Another notable limitation of this study lies in our exclusive inclusion of surgically confirmed recurrence cases, which may introduce potential selection bias. While such rigorous criteria enhance diagnostic specificity, they may systematically exclude subclinical recurrence patients who did not undergo reoperation (e.g., those opting for conservative management or with surgical contraindications), potentially leading to underestimation of true recurrence rates and associated risk factors. Furthermore, reliance on surgical confirmation might obscure the heterogeneous biological characteristics of different recurrence patterns (e.g., local infiltration versus distant metastasis). To address this constraint, we propose that future investigations adopt multimodal diagnostic frameworks integrating dynamic imaging assessments (e.g., contrast-enhanced MRI/PET-CT), liquid biopsy technologies (e.g., ctDNA monitoring), standardized clinical symptom scoring systems, and AI-assisted thyroid nodule diagnosis models, which have demonstrated superior performance in analyzing ultrasound images by identifying subtle morphological features and echogenic patterns often overlooked by human observers (42–44). Such multidimensional validation strategies would not only improve recurrence detection sensitivity but also facilitate the deciphering of molecular evolution patterns in micrometastatic lesions, thereby informing more precise timing for personalized interventions.

In conclusion, this study identifies key recurrence risk factors in DTC using advanced machine learning, enabling personalized clinical strategies. While the ROSE method effectively mitigated class imbalance through synthetic data generation, its use highlights limitations in single-institution datasets with low recurrence rates. To enhance clinical applicability, future work must prioritize larger, multi-institutional cohorts with higher recurrence incidence, reducing reliance on synthetic augmentation and strengthening model generalizability. Cross-institutional collaboration and standardized recurrence monitoring protocols will be critical to validate these models across diverse populations, ensuring equitable integration into global healthcare systems. This approach bridges ML-driven insights with real-world data, advancing precision in DTC management.

## Data Availability

The original contributions presented in the study are included in the article/**Supplementary Material**. Further inquiries can be directed to the corresponding author.
